# The Added Value of a Collagenated Thermosensitive Bone Substitute as a Scaffold for Bone Regeneration

**DOI:** 10.3390/ma17030625

**Published:** 2024-01-27

**Authors:** Charlotte Jeanneau, Jean-Hugues Catherine, Thomas Giraud, Romain Lan, Imad About

**Affiliations:** 1 Aix-Marseille University, CNRS, ISM, 13009 Marseille, Francejean-hugues.catherine@univ-amu.fr (J.-H.C.);; 2 APHM, Hôpital Timone, Pôle Odontologie, Service de Chirurgie Orale, 13005 Marseille, France; romain.lan@univ-amu.fr; 3 Aix-Marseille University, CNRS, EFS, ADES, 13385 Marseille, France

**Keywords:** thermosensitive collagenated bone substitute, bone grafting, stem cell recruitment, angiogenic, osteogenic potential

## Abstract

A pre-hydrated thermosensitive collagenated biomaterial which sets at body temperature and maintains the space of the missing alveolar bone volume, OsteoBiol GTO^®^ (GTO), has been released as a bone substitute. This study was designed to check its angiogenic and osteogenic potentials compared to OsteoBiol Gen-Os^®^ (Gen-Os) and Geistlich Bio-Oss^®^ (Bio-Oss). Samples of materials were incubated in culture media to obtain the extracts. Collagen release was measured in the extracts, which were used to investigate human periodontal ligament (hPDL) cell proliferation (MTT), colonization (Scratch assays) and growth factor release (ELISA). The effects on endothelial cell proliferation (MTT) and organization (Matrigel^®^ assays) were also studied. Finally, endothelial and mesenchymal Stem Cell (hMSC) recruitment (Boyden Chambers) were investigated, and hMSC Alkaline Phosphatase (ALP) activity was measured. A higher collagen concentration was found in GTO extract, which led to significantly higher hPDL cell proliferation/colonization. All materials increased VEGF/FGF-2 growth factor secretion, endothelial cell recruitment, proliferation, and organization, but the increase was highest with GTO. All materials increased hMSC recruitment and ALP activity. However, the increase was highest with collagenated GTO and Gen-Os, which enhanced C5a and BMP-2 secretion. Overall, GTO has higher angiogenic/osteogenic potentials than the collagenated Gen-Os and the anorganic Bio-Oss. It provides a suitable scaffold for endothelial and mesenchymal stem cell recruitment, which represent essential bone regeneration requirements.

## 1. Introduction

After tooth extraction, alveolar bone resorption leads to a soft tissue collapse, which raises major issues for implant placement and the esthetic outcome. Several clinical studies investigated the spontaneous healing and changes in the hard tissues without applying any bone-filling material in the extraction socket. A systematic review including 20 clinical studies reported a significant resorption of the alveolar ridge of 3.8 mm horizontally, and up to 1.24 mm vertically, at 6 months [1].

**Figure 1:**
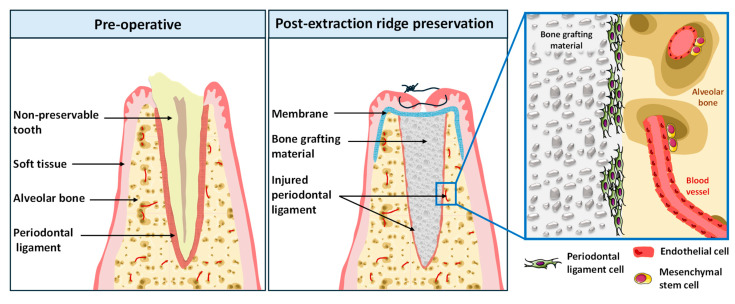
A schematic view of the ridge preservation technique. Tooth extraction leads to a significant alveolar bone resorption. Ridge preservation technique implies the application of bone grafting material in the extraction socket in order to limit the resorption process. Upon its application, the material interacts with the injured periodontal ligament as well as with the endothelial cells and mesenchymal stem cells. The interactions between these actors play a major role in alveolar bone regeneration.

Strategies to overcome this resorption with the aim of preserving alveolar bone have been developed and recognized as alveolar ridge preservation techniques (Figure 1). These are mainly based on the application of bone filling materials immediately in the extraction socket. Several allogenic, xenogenic and synthetic bone substitutes including calcium phosphates, bioceramics, or polymers have been used, but no complete preservation of the socket has been achieved [2,3,4]. Indeed, a recently published systematic review including 88 randomized clinical trials, where 1740 sockets underwent alveolar ridge preservation with different materials, showed that all materials significantly reduced horizontal and vertical shrinkage after tooth extraction compared to spontaneous healing [4,5,6,7].

Collagenated bone substitutes hold promise in reducing the alveolar bone resorption. During the healing process, collagen enhances neovascularization by providing support to the adhesion of endothelial cells through interaction of endothelial cell α2β1integrin binding sites with collagen RGD sequences. This leads to the development of a new vascular network within the material, as demonstrated in vitro [8,9] and in histological studies in vivo [10]. Additionally, collagen hydrolysis and release during the material remodeling process provides a chemotactic gradient, which plays a pivotal role in the recruitment of human bone marrow mesenchymal stem cells (hMSC) by haptotaxis to the bone resorption site to synthesize the new bone [11]. Moreover, the remaining unhydrolyzed collagen after the resorption process and the collagen produced during the bone formation provide nucleation sites for the mineralization of newly formed bone [12]. Moreover, the material’s interaction with the injured human periodontal ligament (hPDL) cells plays a major role in the alveolar bone regeneration process. Indeed, collagenated materials’ interaction with hPDL cells leads to the release of growth factors such as Vascular Endothelial Growth Factor (VEGF) [9], which have been reported to adsorb on the material surface. When the activity of growth factor-impregnated granules was investigated after implantation in critical-size defects in rat calvaria, bone tissue formation and markers for bone and vascularization significantly increased [13]. Also, the interaction of injured hPDL cells with the collagenated bone substitute has been demonstrated to induce the release of the Complement C5a bioactive molecule. This molecule has specific receptors on bone marrow mesenchymal stem cells. Chemotaxis studies performed in vitro clearly demonstrated that this molecule provides a chemotactic gradient for bone marrow mesenchymal stem cells’ recruitment to the material application site to initiate bone regeneration. A previous work comparing collagenated to anorganic bone substitutes in vitro reported higher angiogenic and osteogenic potentials of collagenated materials compared to anorganic bone grafting material [14].

Most collagenated and non-collagenated bone grafting materials are presented in the form of granules, which may collapse after their application in the extraction socket, leading to a partial decrease in alveolar bone regeneration, which, by definition, is limited to the grafting material application site. Thus, maintaining the volume/shape of the extraction socket appears to be key for socket ridge preservation.

A pre-hydrated collagenated heterologous biomaterial coupled with a thermosensitive gel has been released recently: OsteoBiol GTO^®^ (GTO). The material is made up of 80% xenogenic collagenated cortico-cancellous granules, ranging in size from 600 to 1000 µm and blended with 20% TSV Gel. This hydrogel, which contains type I and III collagen with polyunsaturated fatty acids and a thermosensitive synthetic copolymer, is sticky at room temperature and easily adaptable to the recipient site. Upon application at body temperature, it jellifies, and thus provides dimensional stability, leading to easier handling and to maintaining the space which pre-figures the new bone volume. At the same time, it limits the migration of gingival cells into the socket.

**Figure 2:**
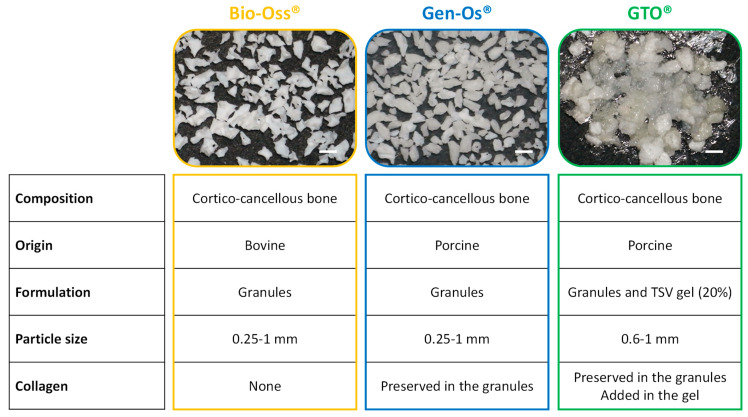
Presentation of the bone grafting materials used. Scale bar = 1 mm.

To our knowledge, no studies have been conducted on this material’s use in the context of ridge preservation in vitro or in vivo. This study was set to check GTO’s angiogenic and osteogenic potentials compared to other xenogenic collagenated OsteoBiol Gen-Os^®^ (Gen-Os) and the anorganic grafting material Geistlich Bio-Oss^®^ (Bio-Oss). Some properties of these materials are reported (Figure 2).

An in vitro model simulating the clinical situation of post-extraction ridge preservation was applied in this study using injured human periodontal ligament cells (Figure 1). This model was used to study the collagenated thermosensitive substitute effects on hPDL cell proliferation/colonization and its angiogenic/osteogenic potentials.

## 2. Materials and Methods

### 2.1. Reagent

All cell culture media and reagents were purchased from Dominique Dutscher (Brumath, France). GTO^®^ (GTO) and Gen-Os^®^ (Gen-Os) of OsteoBiol^®^ series for bone substitutes were obtained from Tecnoss^®^ Dental s.r.l., Turin, Italy), and Geistlich Bio-Oss^®^ (Bio-Oss) material was from Geistlich Pharma (Wolhusen, Switzerland).

### 2.2. Cell Culture

Human periodontal ligament cells were prepared from sound third molars freshly extracted for orthodontics reasons in compliance with French legislation and Aix-Marseille University ethical committee agreement (N/Ref: 2022-05-12-003). Briefly, after the extraction process, teeth were washed, and the periodontal ligament mechanically removed by scraping the middle third of the root surface with a sterile curette. The extirpated dental ligament was minced, and explants were cultured in 100-mm-diameter culture dishes. Confluent cultures were collected by trypsinization and subcultured. The cells were cultured in minimum essential medium (MEM) supplemented with 10% fetal bovine serum, 2 mM glutamine, 100 UI/mL penicillin, 100 µm streptomycin, and 0.25 µg/mL amphotericin B at 37 °C in a 95% air plus 5% CO_2_ atmosphere.

Human Mesenchymal Stem Cells from Bone Marrow (hMSC) and Human Umbilical Vein Endothelial Cells (HUVEC) were purchased from PromoCell (Heidelberg, Germany) and routinely cultured in an optimized PromoCell media, respectively, while MSCs Growth Medium 2 (MGM-2) and Endothelial Cell Growth Medium 2 (EGM-2) were kept at 37 °C in a 95% air plus 5% CO_2_.

### 2.3. Bone-Filling Material Extracts

**Figure 3:**
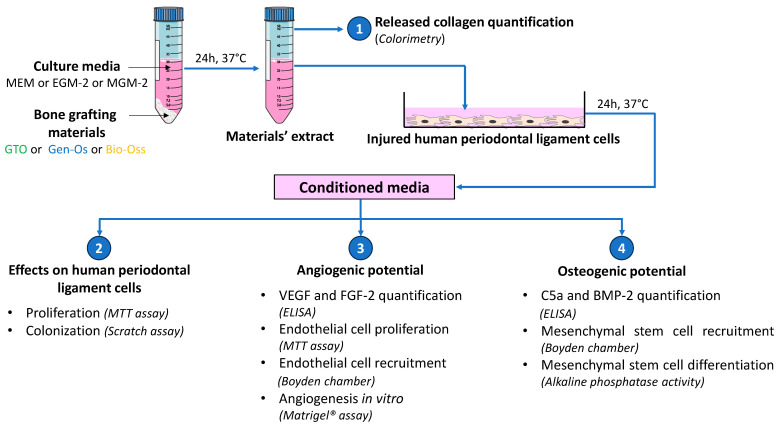
A schematic view of the experimental setup. The materials’ extracts were obtained by incubating the bone-filling materials in the respective cell culture media (24 h, 37 °C, 5% CO_2_). The released collagen was measured in the extracts by quantifying hydroxyproline using a colorimetric method. Physically injured hPDL cells were incubated with the obtained extracts for 24 h. The supernatants from injured hPDL cells (conditioned media) were applied on intact hPDL cells to evaluate their proliferation using the MTT test. hPDL colonization of the injured site was studied using the scratch assay. VEGF and FGF-2 secretion in the conditioned media was quantified by ELISA. Conditioned media were applied on endothelial cells (HUVEC) to investigate their proliferation using MTT test, their angiogenic potential using Matrigel^®^ assay and their recruitment in Boyden chambers. Finally, C5a and BMP-2 secretion in the conditioned media was quantified by ELISA. Conditioned media were applied on hMSC cultures to quantify their migration using Boyden chambers, and to evaluate their osteogenic potential using alkaline phosphatase assay.

To obtain bone-filling materials’ extracts, each material was incubated separately in each of the 3 culture media without serum. Depending on the type of experiment, the extracts were prepared in: MEM for hPDL culture; in MGM-2 for mesenchymal stem cell culture or EGM-2 for endothelial cell culture at 20 mg/mL (37 °C, 24 h). Samples were centrifuged and the supernatants were collected. The supernatants containing the materials’ extracts were used for the next experimental protocols, as demonstrated (Figure 3).

### 2.4. The Measurement of Collagen Released in the Materials’ Extracts

The concentration of collagen was quantified in the extracts obtained in MEM culture medium using a commercially available kit based on the detection of hydroxyproline using a colorimetric method (The QuickZyme Total Collagen assay, QuickZyme Biosciences B.V., Leiden, The Netherlands) by measuring the absorbance at 570 nm using a spectrophotometer (Σ960, MeterTech, Taipei, Taiwan).

### 2.5. Conditioned Media Preparation

Subconfluent hPDL cell cultures in 6-well plates were washed, injured with a sterile scalpel (5 horizontal and 5 vertical direction lanes) and incubated with 2 mL of material extract (20 mg/mL) or serum-free control media. After 24 h, the supernatants were harvested and referred to as “Conditioned media” for the next experiments. Depending on the experiment, conditioned media were prepared in MEM, MGM-2 or EGM-2 medium.

### 2.6. Cell Proliferation

HUVEC or hPDL cells were cultured at a low density (1000 cells/cm^2^) in 96-well culture plates. Media were replaced by conditioned media. After 3, 6, and 9 days, the supernatants were removed and MTT assay was performed. Medium were removed and immediately replaced with 1 mL/well of a 0.5% of 3-(4,5-dimethylthiazol-2-yl)-2,5-diphenyl tetrazolium bromide). After incubation for 2 h at 37 °C, the supernatant was discarded, and the formazan crystals solubilized with 100 µL/well of dimethyl sulfoxide (DMSO). Conditioned media without material extracts were used as a control. The absorbance of each 6-well dish was measured using an automatic microplate spectrophotometer (Σ960) at 550 nm, and results were expressed as percentages of control (conditioned MEM for hPDL cells and conditioned EGM-2 for endothelial cells).

### 2.7. Scratch Wound Healing Assay

The effect of the materials on hPDL cells’ colonization was evaluated using the Scratch Wound Healing Assays. Confluent hPDL cells (30,000 cells/cm^2^) grown in 6-well plates were injured with a pipette tip and incubated with conditioned medium. Conditioned MEM media without extracts were used as a control. After 12 h of incubation, cell nuclei were labelled with 1 mg/mL DAPI (20 min), and migrating cells to the injured zone were counted in 5 random fields under a Carl Zeiss Axiovert200 fluorescence microscope (Carl Zeiss S.A.S., Marly Le Roi, France). Results are expressed as percentages of control (conditioned MEM medium).

### 2.8. Osteogenic/Angiogenic Growth Factor and C5a Complement Protein Quantification

The secretion of VEGF, Fibroblast Growth Factor 2 (FGF-2), Bone Morphogenic Protein 2 (BMP-2) and C5a in conditioned media were quantified by the enzyme-linked immunosorbent assay using the Duoset human kits (R&D Systems, Minneapolis, MN, USA) according to the manufacturer’s instructions.

### 2.9. Cell Recruitment

The cell migration assays were performed in 12-well plates (lower chamber) fitted with 8 mm diameter Boyden inserts (upper chamber). Confluent hPDL cells were cultured in the lower chamber. After washing with MEM, they were injured and incubated either in MEM (control) or with material extract (1 mL). The upper chambers were seeded with HUVEC or hMSCs in 100 µL MEM medium (10^4^ cells/well). After 24 h, migrating cells on the lower side of the membrane were fixed (15 min, cold ethanol 70%) and then labelled (20 min, DAPI). Migrating cells on the lower side of the membrane were counted on 5 random fields using fluorescence microscopy. Results are expressed as percentages of control (cells migrating in response to injured hPDL cells incubated in MEM serum-free medium).

### 2.10. Neoangiogenesis

For the assessment of the angiogenic capacity, HUVECs (4 × 10^5^ cells/well) were seeded on Matrigel^®^ extracellular matrix and cultured in the conditioned media. The endothelial cell organization was observed with a phase-contrast microscope (Carl Zeiss Axiovert200) after 24 h of culturing. Angiogenic capacity was quantitatively evaluated by counting the number of closed structures. Results are expressed as percentages of control (conditioned EGM-2).

### 2.11. Mesenchymal Stem Cell ALP Activity

Osteogenic differentiation of hMSCs was studied by quantifying Alkaline Phosphatase (ALP) enzyme activity using a colorimetric ALP Kit (Abcam, Cambridge, UK). hMSCs (10^5^ cells) seeded in 6-well plates were incubated with conditioned media for 7 days. Upon reaching confluency, the cells were dissociated using trypsin and counted. After rinsing, they were collected using 50 µL/10^5^ cell lysis buffer and three-time 10-s ultra-sonic baths. Samples were centrifuged at 4 °C for 15 min to remove any insoluble material, and the supernatant was collected and kept on ice. Samples were placed in a 96-well plate and ALP measurement was performed according to the manufacturer’s instructions. Absorbance was measured at OD 405 nm with a microplate reader (Σ960). Results are expressed as the percentage of control (conditioned MGM-2).

### 2.12. Statistical Analysis

All experiments were repeated at least three times on three different cell populations, and statistical significance (*p* < 0.05) was determined using the Student’s *t*-test to compare the different treatments and their respective controls.

## 3. Results

### 3.1. Collagen Is Released in Collagenated Materials’ Extracts

**Figure 4:**
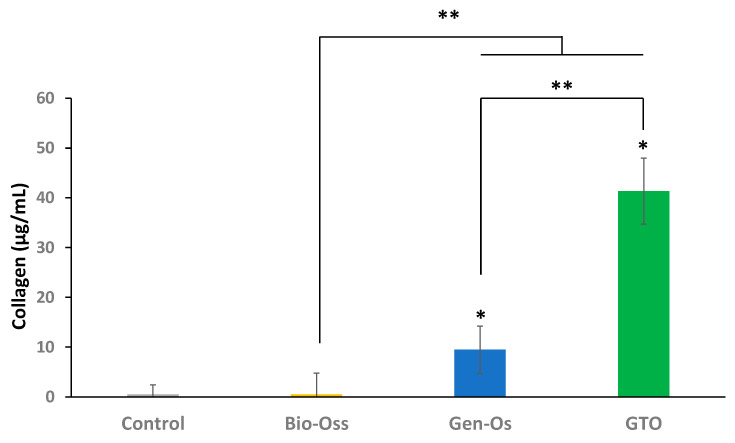
Measurement of collagen release in the extracts. A significant release of collagen was detected in the collagenated Gen-Os and GTO extracts. The released collagen concentration from GTO was four times higher than that with Gen-Os. Unsurprisingly, collagen was not detected in the anorganic material extract. * Significant differences with the control (MEM); ** significant differences between two conditions (*p* < 0.05).

Collagen was detected in the extracts of collagenated materials, but not in the anorganic Bio-Oss extract. Its concentration in GTO extract was four times higher than in the Gen-Os extract (Figure 4).

### 3.2. The Effects of the Materials on Human Periodontal Ligament Cells

#### 3.2.1. Bone Grafting Materials Increased hPDLs’ Cell Proliferation

**Figure 5:**
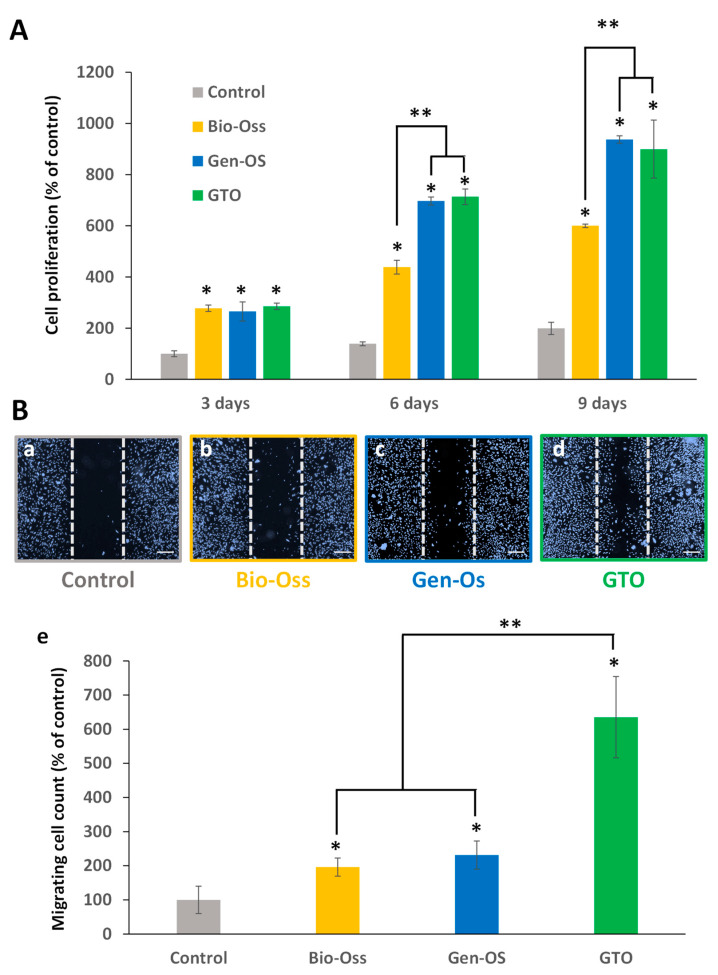
(**A**) Effects of the materials on hPDL cell proliferation. All materials significantly increased cell proliferation after 3, 6 and 9 days compared with the control (conditioned MEM). However, from day 6, the increase with Gen-Os and GTO was significantly higher than with Bio-Oss. (**B**) Scratch wound healing assays of hPDL cells cultured for 24 h with conditioned media. (**a**–**d**) Representative pictures taken with a fluorescence microscope: (**a**) control, (**b**) Bio-Oss, (**c**) Gen-Os, and (**d**) GTO. The two vertical dashed lines delineate the initial cell-free zone at the injury site, and cells migrating to colonize this area were counted. Scale bars = 500 µm. (**e**) Number of migrating cells to the injury area significantly increased with all materials, but to a higher extent with GTO. Results are expressed as percentages of the control (conditioned MEM). * Significant differences with the control; ** significant differences between two conditions (*p* < 0.05).

The proliferation of hPDL cells significantly increased with the three bone grafting materials at 3, 6 and 9 days compared to the control. Cell proliferation with Gen-Os and GTO was significantly higher than that with Bio-Oss at 6 and 9 days (Figure 5A).

#### 3.2.2. Bone Grafting Materials Increased hPDLs’ Cell Colonization

In the scratch assay, all materials significantly increased the hPDL cell colonization of the wound (cell-free zone) compared to the control (conditioned MEM). However, this increase was significantly higher with GTO than with Bio-Oss or Gen-Os (Figure 5B).

### 3.3. Angiogenic Potential

#### 3.3.1. Bone Grafting Materials Increased Angiogenic Growth Factors’ Secretion by hPDL Cells

**Figure 6:**
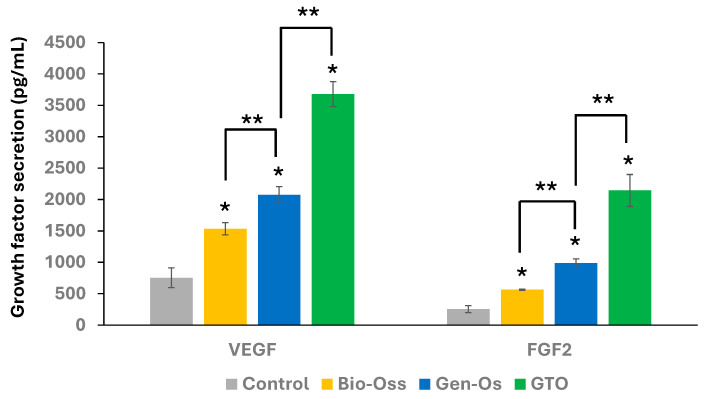
Angiogenic growth factor secretion by hPDL cells. ELISA measurements showed that all materials significantly increased VEGF and FGF-2 secretion compared to the control (Conditioned MEM), but the increase was highest with GTO. Results are expressed in pg/mL. * Significant differences with the control; ** significant differences between two conditions (*p* < 0.05).

Although the secretion of VEGF and FGF-2 increased with all materials, the increase with the collagenated materials was higher than with Bio-Oss. The highest increase was observed with GTO (Figure 6).

#### 3.3.2. Bone Grafting Materials Increased Endothelial Cell Proliferation

**Figure 7:**
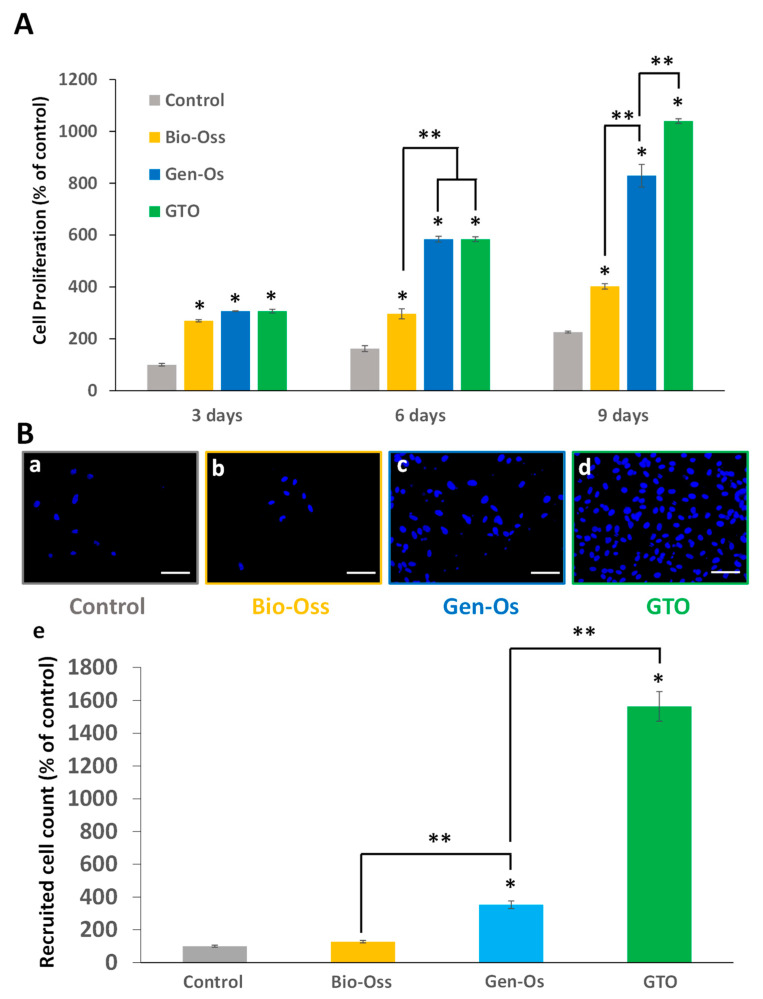
(**A**) Effects of the materials on HUVEC proliferation. All materials significantly increased cell proliferation compared to the control (conditioned EGM-2). However, the increase was significantly higher with Gen-Os and GTO than with Bio-Oss at day 6 and 9. (**B**) Effects of materials on HUVEC migration in Boyden Chambers. (**a**–**d**) Representative pictures used for migrating cell count. (**a**) Control, (**b**) Bio-Oss, (**c**) Gen-Os, and (**d**) GTO (scale bars = 50 μm). (**e**) Data analysis showed that only Gen-Os and GTO significantly increased HUVEC recruitment when compared to the control or to the anorganic Bio-Oss. This increase was four times higher with GTO than with Gen-Os. Results are expressed in percentages of the control (conditioned EGM-2). * Significant differences with the control; ** significant differences between two conditions (*p* < 0.05).

A significant increase in HUVEC proliferation was obtained with all tested materials. The proliferation with Gen-Os and GTO was significantly higher than with Bio-Oss from day 6. The increase with GTO was higher than that with Gen-Os at day 9 (Figure 7A).

#### 3.3.3. Collagenated Materials Increased Endothelial Cell Recruitment

A significant increase in HUVEC recruitment was observed with both collagenated materials Gen-Os and GTO compared to the control (conditioned MEM) or to Bio-Oss. The increase with GTO was four times higher than that with Gen-Os (Figure 7B).

#### 3.3.4. Bone Grafting Materials Increased Tube-like Structure Formation

**Figure 8:**
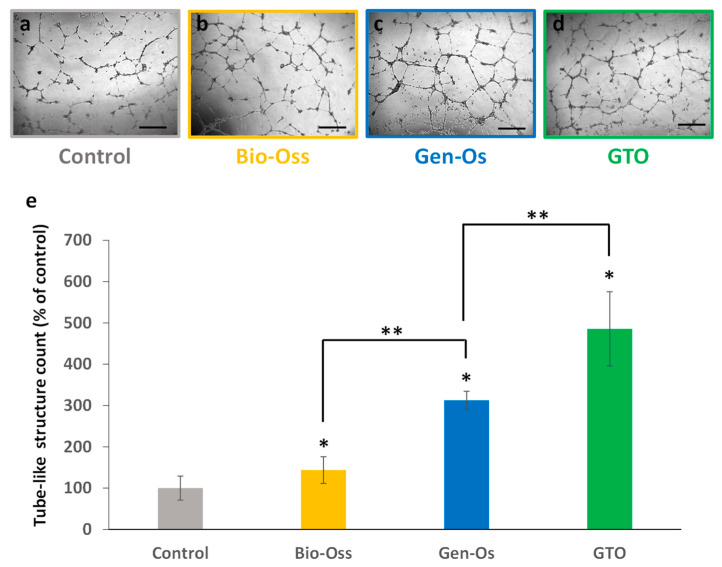
Effect of bone grafting materials on neoangiogenesis in vitro. When HUVEC were incubated with the conditioned media, they organized into closed structures and formed a capillary-like network. (**a**–**d**) Representative pictures taken with an optical microscope: (**a**) control (conditioned EGM-2), (**b**) Bio-Oss, (**c**) Gen-Os, and (**d**) GTO (scale bars = 50 µm). (**e**) The number of closed tube-like structures significantly increased with all materials, but the increase was higher with GTO. Results are expressed in percentages of the control (conditioned EGM-2). * Significant differences with the control; ** significant differences between two conditions (*p* < 0.05).

After endothelial cell culture on Matrigel^®^ (Corning, NY, USA) for 24 h, the formation of capillary-like closed structures of well-organized endothelial cells was observed with all materials and with the control (conditioned EGM-2). Among the three materials, collagenated substitutes were more potent and GTO induced the highest number of tube-like structures (Figure 8).

### 3.4. Osteogenic Potential

#### 3.4.1. Collagenated Materials Enhanced C5a and BMP-2 Secretion by hPDL Cells

**Figure 9:**
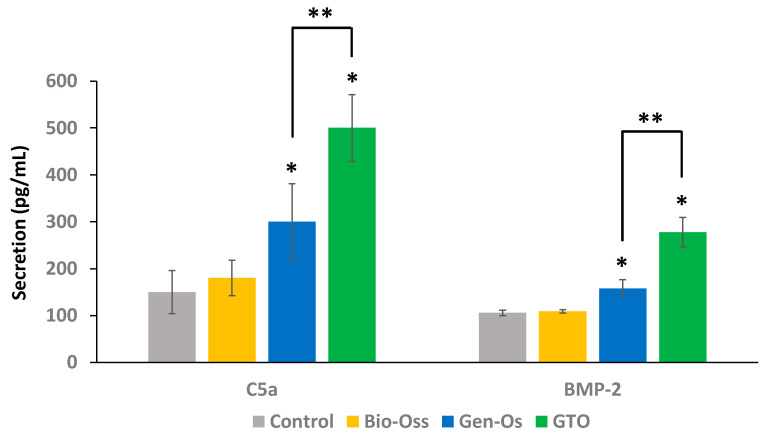
Complement C5a molecule and BMP-2 growth factor secretion by hPDL cells. ELISA showed that Gen-Os and GTO significantly increased C5a and BMP-2 secretion when compared with the control (conditioned MEM) and to Bio-Oss. This increase was significantly higher with GTO. Results are expressed in pg/mL. * Significant differences with the control; ** significant differences between two conditions (*p* < 0.05).

Gen-Os and GTO significantly increased C5a and BMP-2 secretion when compared to the control (conditioned MEM) and to Bio-Oss. The increase induced by GTO was significantly higher than with Gen-Os (Figure 9).

#### 3.4.2. Bone Grafting Materials Enhanced Mesenchymal Stem Cell Recruitment

**Figure 10:**
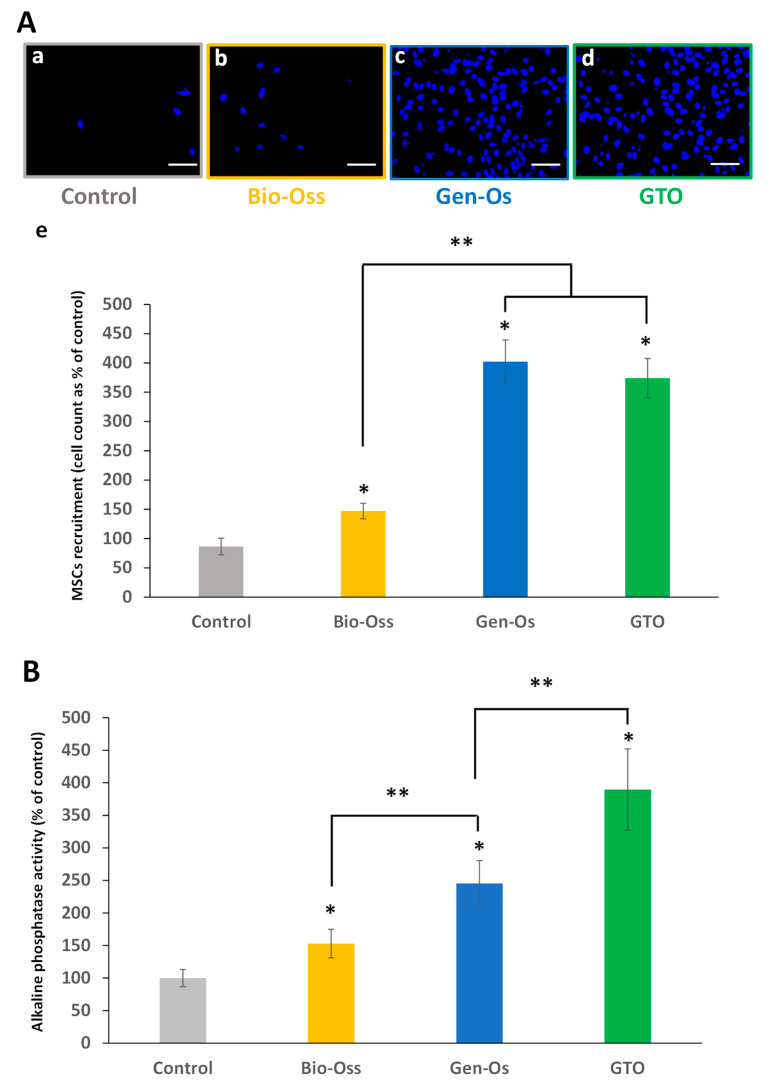
(**A**) Effects of the materials on hMSC migration in Boyden Chambers. (**a**–**d**) Representative pictures used for migrating cell count. (**a**) Control (conditioned MEM), (**b**) Bio-Oss, (**c**) Gen-Os, and (**d**) GTO (scales bars = 50 μm). (**e**) Data show that all materials significantly increased hMSC migration when compared with the control. However, the number of migrating cells was significantly higher with the collagenated Gen-Os and GTO compared to the anorganic Bio-Oss. Results are expressed in percentages of the control. (**B**) Alkaline phosphatase activity measurement. Incubating hMSCs for 7 days with the conditioned media from all materials significantly increased their alkaline phosphatase activity compared to the control (conditioned MGM-2). The increase was significantly higher with Gen-Os and GTO than with Bio-Oss. The increase of alkaline Phosphatase activity with GTO was significantly higher than with Gen-Os. Results are expressed in percentages of the control. * Significant differences with the control; ** significant differences between two conditions (*p* < 0.05).

When hPDL cells seeded in the lower chamber were incubated with the materials’ extracts, a significant number of hMSCs migrated to the lower compartment with all the materials. The increase with collagenated Gen-Os and GTO was significantly higher than with Bio-Oss (Figure 10A).

#### 3.4.3. The Bone Substitutes Induced Mesenchymal Stem Cells’ Alkaline Phosphatase Activity

All materials significantly increased hMSC ALP activity compared to the control (conditioned MGM-2). A significant increase was observed when hMSCs were incubated with collagenated GTO and Gen-Os when compared to anorganic Bio-Oss. The increase with GTO was significantly higher than with Gen-Os (Figure 10B).

## 4. Discussion

This study demonstrated that the pre-hydrated thermosensitive collagenated GTO bone substitute has significant angiogenic and osteogenic potentials, and that it could be used as a suitable scaffold for the recruitment of both endothelial cells and bone marrow mesenchymal stem cells in bone regeneration.

The interaction of the thermosensitive substitute with injured human periodontal ligament cells leads to a significant secretion of VEGF, FGF-2 and BMP-2 growth factors, as well as the bioactive Complement C5a molecule. This secretion was significantly higher than with the anorganic bone substitute Bio-Oss, and was even higher than that with another collagenated substitute, Gen-Os.

The secretion of these molecules following the interaction of bone substitutes with the target periodontal ligament cells is not a new finding [9,14]. It has been reported that injured human fibroblasts and periodontal ligament cells secrete VEGF and FGF-2 angiogenic factors, which enhance endothelial cell proliferation, adhesion on collagen fibers and the formation of a tube-like network in vitro [9,15]. The mechanism by which VEGF induces endothelial cell adhesion on collagen has been investigated in vitro, and demonstrated that VEGF induces the endothelial surface protein expression of two collagen receptors, the α1β1 and α2β1 integrins, by inducing mRNAs encoding the α1 and α2 subunits. A combination of blocking antibodies to α1 and α2 subunits markedly inhibited VEGF-driven angiogenesis in vitro, and a confirmation was reported in vivo after subcutaneous injection of Matrigel^®^ and VEGF-transfected cells in nude mice [8,16]. This brings an added value to understanding the consequence of both the presence of collagen in the bone substitute and the induction of VEGF secretion upon the interaction of the material with the injured periodontal ligament. Indeed, enhancing the expression of collagen receptors on endothelial cells enhances their adhesion on collagen fibers and neoangiogenesis.

Additionally, BMP-2 has long been established as an important growth factor for bone formation [17], and its expression is essential for bone fracture healing [18] and for embryonic patterning [19]. BMP-2 has been shown to induce osteoblastic differentiation in vitro [20], and to promote bone regeneration in rat critical-sized calvarial defects in vivo [21]. This explains why its use for bone regeneration has been approved by FDA and commercialized (Medtronic®, Minneapolis, MN, USA) as a bone graft material (Infuse^®^) which is composed of recombinant human bone morphogenetic protein-2 and an absorbable collagen sponge carrier. Its use has been reported for interbody spine fusion, fresh tibial fractures, and oral maxillofacial bone-grafting procedures [22].

The combined secretion of BMP-2 with VEGF has been shown to play a synergistic effect in bone regeneration. When mouse muscle-derived stem cells were transduced to express BMP-2, the administration of sFlt1 as a specific antagonist of VEGF significantly inhibited BMP-2-induced bone formation. By contrast, the delivery of exogenous VEGF enhanced BMP-2-induced bone formation and bone healing by improving angiogenesis. This has been demonstrated in ectopic bone formation in mice and critical skull defect healing, as revealed on radiographies and Von Kossa staining on histological sections [23].

The enhanced bone formation seems to be due to the significant recruitment of bone marrow stem cells and endothelial cells. This recruitment is enhanced by the secretion of the Complement C5a bioactive fragment upon the interaction of the collagenated material with the periodontal ligament cells, as demonstrated previously with Gen-Os. Once secreted, C5a has been shown to bind to and to phosphorylate its specific receptor (C5aR) on bone marrow mesenchymal stem cells, leading to their proliferation and recruitment [14]. The expression of C5aR during fractures has also been shown to be involved in osteoblastic differentiation and bone healing in rats [24]. The C5a level obtained with the thermosensitive substitute in this study was higher that what has been demonstrated with anorganic Bio-Oss, and even higher than with the collagenated Gen-Os, which highlights a higher osteogenic potential of the thermosensitive GTO.

Interestingly, the recruitment of both bone marrow mesenchymal stem cells and endothelial stem cells was enhanced with GTO. This highlights that the thermosensitive material provides a suitable scaffold for both endothelial and bone marrow mesenchymal stem cells, which are required for bone regeneration. Indeed, a previous investigation reported that mesenchymal stem cells release VEGF and FGF-2, which adsorb on the collagenated Gen-Os granules. The granules with adsorbed growth factors enhanced the osteogenic commitment by increasing runt-related transcription factor 2 (RUNX2), osteopontin, osteonectin, osteocalcin, and collagen type I osteogenic markers, compared to the native bone granules. This was also associated with a marked increase in the expression of the endothelial cell markers (CD31, von Willebrand Factor, and VEGF) in the granules with adsorbed growth factors. When these granules, with adsorbed growth factors, were engrafted in rat calvaria bone defects, they enhanced angiogenesis and new bone formation [13].

Surprisingly, the growth factors’ secretion level obtained with GTO upon its interaction with human periodontal ligament cells was higher than with Gen-Os, although the granules of both materials contain collagen. Similarly, the endothelial cell proliferation and angiogenic potentials were higher with GTO. Additionally, the recruitment of endothelial cells and mesenchymal stem cells were significantly higher with GTO. This appears surprising, and one may wonder where the difference comes from. GTO is composed of 80% collagenated cortico-cancellous granules ranging in size from 600 to 1000 µm, properly blended with 20% TSV Gel containing type I and type III collagen. Gen-Os is made of collagenated cortico-cancellous granules (250–1000 µm). Although the company does not report the concentration of collagen in the hydrogel, we wondered if this might provide an explanation for the observed results in our study. For this purpose, we investigated the presence of hydroxyproline, which can be used as a direct measure of the total amount of collagen. This amino acid is limited to the triple helix of collagen, where its presence increases the triple helix stability. While no collagen was detected in the anorganic Bio-Oss supernatant, a significant concentration was detected in Gen-Os supernatant, and collagen secretion with GTO was four times higher than with Gen-Os.

This is an important result, as it provides an interpretation of the results obtained here, and particularly in explaining the difference between the collagenated Gen-Os and GTO. Indeed, while both materials contain native collagen in the granules, GTO contains an additional quantity of collagen in the hydrogel which is readily available. Indeed, a significant concentration of collagen (40 µg/mL) was present in the GTO supernatant after 24 h. The presence of this collagen has been demonstrated to play a major role in providing a support for the adhesion of endothelial cells and neoangiogenesis, as well as for recruiting bone marrow mesenchymal stem cells.

Indeed, as reported in many studies, collagen fibers provide adhesion sites for endothelial cell integrins, leading to the adhesion of endothelial cells to collagen fibers and subsequent neovascularization [8,16]. Also, collagen hydrolysis during the resorption process provides a chemotaxis gradient for bone marrow mesenchymal stem cells, which are recruited to the bone substitute application site by haptotaxis, where they differentiate into osteoblastic cells to synthesize the new bone.

During bone remodeling, the majority of the newly recruited osteoblast lineage cells positioned immediately next to the osteoclasts exhibit uPARAP/Endo180, an endocytic collagen receptor reported to be involved in collagen internalization and cell migration in various cell types. The inactivation of this receptor is reported to inhibit bone formation, leading to skeletal deformities. The use of antibodies directed against this receptor in vitro inhibited collagen internalization in an osteoblastic lineage, and decreased their migration to the surface-bound collagen in the transmembrane migration assay [11]. Cell migration induced by changes in the extracellular matrix (ECM) have also been shown in other tissues [9,25], and are called haptotaxis when the signal is ECM-bound, in contrast with chemotaxis, when the signal is a soluble factor [26,27]. Accordingly, insoluble collagen was reported to induce the haptotaxis of human and rabbit MSCs [11,26].

The fact that our experiments were conducted using a single concentration of the materials and only at one time period may underestimate the quantities/number of factors that may be released at later time periods. Additionally, while only the extracts were used in this study, further investigations into the direct interaction of the materials with the cells will add to our understanding on the materials’ effectiveness in bone regeneration.

Studies on the thermosensitive GTO are scant. The only study evaluating GTO effects in vitro was conducted on STRO-1 sorted human dental pulp mesenchymal stem cells (hDPSCs) and investigated its biocompatibility compared to two other xenogenic bone substitutes, Gen-Os and OsteoBiol^®^ Apatos^®^, by evaluating their effects on cell morphology, adhesion, and the proliferation of hDPSCs. The study reported that the three biomaterials were biocompatible. Interestingly, when hDPSCs were incubated with the collagenated GTO and Gen-Os, an induction of intracellular osteopontin and an enhanced alkaline phosphatase activity were observed, suggesting that these materials contribute to the osteogenic commitment of these cells [28].

A single clinical study aiming at investigating GTO was conducted on its effectiveness of transcrestal maxillary sinus augmentation using different heterologous bone substitutes in 75 sinus-grafting procedures, where 89 implants were placed in 66 patients. Clinical and radiographical evaluations were performed over a follow-up period of 93 ± 55 months. The study reported that none of the implants were lost during the observation period [29].

## 5. Conclusions

Taken together, these data show that the thermosensitive collagenated bone substitute has higher angiogenic and osteogenic potentials than the anorganic substitute, and even higher than the collagenated Gen-Os.

While no studies were performed using GTO for alveolar ridge preservation, the present study sets the basic foundations for its use as a scaffold for bone filling in oral surgery, and in particular for alveolar ridge preservation. Indeed, the thermosensitive GTO responds to the criteria required for ridge preservation, as it jellifies at body temperature and, thus, keeps the volume required for the new bone formation. Also, it provides a suitable scaffold for the endothelial and stem cell recruitment which are required for new bone tissue. Overall, this work highlights the added value of a collagenated thermosensitive bone substitute as a scaffold for bone regeneration.

## Data Availability

Data are contained within the article.
